# Supplementary material for: Impact of COVID-19 pandemic on surgical neuro-oncology multi-disciplinary team decision making: a national survey (COVID-CNSMDT Study)
Source: BMJ Open. 2020 Aug 16;10(8):e040898. doi: 10.1136/bmjopen-2020-040898 (PMC7430412; doi:10.1136/bmjopen-2020-040898)
Supplement: Author's manuscript [file bmjopen-2020-040898.draft_revisions.pdf]

BMJ Open

BMJ Open is committed to open peer review. As part of this commitment we make the peer review history of every article we publish publicly available.

When an article is published we post the peer reviewers' comments and the authors' responses online. We also post the versions of the paper that were used during peer review. These are the versions that the peer review comments apply to.

The versions of the paper that follow are the versions that were submitted during the peer review process. They are not the versions of record or the final published versions. They should not be cited or distributed as the published version of this manuscript.

BMJ Open is an open access journal and the full, final, typeset and author-corrected version of record of the manuscript is available on our site with no access controls, subscription charges or pay-per-view fees (http://bmjopen.bmj.com).

If you have any questions on BMJ Open's open peer review process please email info.bmjopen@bmj.com

BMJ Open

Impact of COVID-19 Pandemic on Surgical Neuro-oncology Multi-Disciplinary Team Decision Making (COVID-CNSMDT Study)

Journal:	*BMJ Open*
Manuscript ID	bmjopen-2020-040898
Article Type:	Original research
Date Submitted by the Author:	25-May-2020

Complete List of Authors:	Price, Stephen; Cambridge University, Neurosurgery Division, Dept. Clinical Neurosciences<br>Joannides, Alexis; Cambridge University, Neurosurgery Division, Department of Clinical Neurosciences<br>Plaha, Puneet; John Radcliffe Hospital, Department of Neurosurgery<br>Afshari, Fardad; University Hospitals Coventry and Warwickshire NHS Trust, Department of Neurosurgery<br>Albanese, Ermina; University Hospitals of North Midlands NHS Trust, Department of Neurosurgery<br>Barua, Neil; North Bristol NHS Trust, Department of Neurosurgery<br>Chan, Huan Wee; University Hospital Southampton NHS Foundation Trust, Department of Neurosurgery<br>Critchley, Giles; Brighton and Sussex University Hospitals NHS Trust, Department of Neurosurgery<br>Flannery, Thomas; Royal Victoria Hospital<br>Fountain, Daniel; Salford Royal NHS Foundation Trust, Manchester Centre for Clinical Neurosciences<br>Mathew, Ryan; Leeds General Infirmary, Department of Neurosurgery<br>Piper, Rory; John Radcliffe Hospital, Department of Neurosurgery<br>Poon, Michael; The University of Edinburgh, Usher Institute<br>Rajaraman, Chittoor; Hull Royal Infirmary, Department of Neurosurgery<br>Rominiyi, Ola; Royal Hallamshire Hospital, Department of Neurosurgery<br>Smith, Stuart; Queen's Medical Centre, Department of Neurosurgery<br>Solomou, Georgios; University Hospitals Coventry and Warwickshire NHS Trust, Department of Neurosurgery; University of Birmingham College of Medical and Dental Sciences<br>Solth, Anna; Ninewells Hospital, Department of Neurosurgery<br>Surash, Surash; Royal Victoria Infirmary, Department of Neurosurgery<br>Wykes, Victoria; Queen Elizabeth Hospital, Department of Neurosurgery<br>Bulbeck, Helen; brainstrust<br>Hutchinson, Peter; Cambridge University, Academic Neurosurgery Division, Department of Clinical Neurosciences<br>Jenkinson, Michael; The Walton Centre NHS Foundation Trust, Department of Neurosurgery

Keywords:	Neurological oncology < ONCOLOGY, Change management < HEALTH SERVICES ADMINISTRATION & MANAGEMENT, INFECTIOUS DISEASES

I, the Submitting Author has the right to grant and does grant on behalf of all authors of the Work (as defined in the below author licence), an exclusive licence and/or a non-exclusive licence for contributions from authors who are: i) UK Crown employees; ii) where BMJ has agreed a CC-BY licence shall apply, and/or iii) in accordance with the terms applicable for US Federal Government officers or employees acting as part of their official duties; on a worldwide, perpetual, irrevocable, royalty-free basis to BMJ Publishing Group Ltd ("BMJ") its licensees and where the relevant Journal is co-owned by BMJ to the co-owners of the Journal, to publish the Work in this journal and any other BMJ products and to exploit all rights, as set out in our licence.

The Submitting Author accepts and understands that any supply made under these terms is made by BMJ to the Submitting Author unless you are acting as an employee on behalf of your employer or a postgraduate student of an affiliated institution which is paying any applicable article publishing charge ("APC") for Open Access articles. Where the Submitting Author wishes to make the Work available on an Open Access basis (and intends to pay the relevant APC), the terms of reuse of such Open Access shall be governed by a Creative Commons licence – details of these licences and which Creative Commons licence will apply to this Work are set out in our licence referred to above.

Other than as permitted in any relevant BMJ Author's Self Archiving Policies, I confirm this Work has not been accepted for publication elsewhere, is not being considered for publication elsewhere and does not duplicate material already published. I confirm all authors consent to publication of this Work and authorise the granting of this licence.

For peer review only

Impact of COVID-19 Pandemic on Surgical Neuro-oncology Multi-Disciplinary Team Decision Making (COVID-CNSMDT Study)

Stephen J Price[1], Alexis J Joannides[1], Puneet Plaha[2], Fardad T Afshari[3], Erminia Albanese[4], Neil U Barua[5], Huan Wee Chan[6], Giles Critchley[7], Thomas Flannery[8], Daniel M Fountain[9], Ryan Mathew[10], Rory J Piper[2], Michael TC Poon[11], Chittoor Rajaraman[12], Ola Rominiyi[13], Stuart Smith[14], Georgios Solomou[3,15], Anna Solth[16], Surash Surash[17], Victoria Wykes[18], Helen Bulbeck[19], Peter Hutchinson[1] and Michael D Jenkinson[20]

On behalf of the COVID-CNSMDT study group

[1]Neurosurgery Division, Dept. Clinical Neurosciences, University of Cambridge, Cambridge CB2 0QQ; [2] Dept of Neurosurgery, John Radcliffe Hospital, Oxford OX3 9DU; [3] Dept of Neurosurgery, University Hospital Coventry, CV2 2DX; [4]Dept. of Neurosurgery, University Hospital of North Midlands, Stoke on Trent, ST46QG; [5]Dept. of Neurosurgery, North Bristol NHS Trust, Bristol BS10 5NB; [6]Dept of Neurosurgery, Wessex Neurological Centre, Southampton SO16 6YD; [7]Dept of Neurosurgery, Brighton and Sussex University Hospitals NHS Trust, Brighton BN2 5BE; [8]Dept of Neurosurgery, Royal Victoria Hospital, Belfast BT12 6BA; [9] Manchester Centre for Clinical Neurosciences, Salford Royal NHS Foundation Trust, Salford M6 8HD, [10]Dept of Neurosurgery, General Infirmary at Leeds, Leeds LS1 3EX; [11]Usher Institute, University of Edinburgh, EH16 4SB; [12]Dept of Neurosurgery, Hull Royal Infirmary, Hull HU3 2JZ; [13]Dept of Neurosurgery, Royal Hallamshire Hospital, Sheffield S10 2JF; [14]Dept of Neurosurgery, Queen's Medical Centre, Nottingham NG7 2UH; [15]University of Birmingham, Birmingham B15 2TT; [16]Dept of Neurosurgery, Ninewells Hospital and Medical

For peer review only - http://bmjopen.bmj.com/site/about/guidelines.xhtml

School, Dundee DD1 9SY; [17]Dept of Neurosurgery, Royal Victoria Infirmary, Newcastle upon Tyne NE1 4L; [18]Dept of Neurosurgery, Queen Elizabeth Neuroscience Centre, Birmingham B15 2TB; [19]Brainstrust, Cowes, Isle of Wight PO31 7QG; [20]Dept of Neurosurgery, University of Liverpool & The Walton Centre NHS Trust, Liverpool, L9 7LJ.

Corresponding Author:

Mr Stephen J Price
Hon. Consultant Neurosurgeon
Department of Clinical Neurosciences,
Division of Neurosurgery,
Box 167 Cambridge Biomedical Campus,
Cambridge CB2 0QQ.

E-mail: sjp58@cam.ac.uk
Tel: 01223 746455

WORD COUNT: 1485 words

For peer review only

For peer review only - http://bmjopen.bmj.com/site/about/guidelines.xhtml

ABSTRACT:

Objectives: Pressures on healthcare systems due to COVID-19 has impacted non-COVID patients with surgery disproportionally affected. This study aims to understand the impact on the initial management of patients with brain tumours by measuring changes to normal multidisciplinary team (MDT) decision making.

Design: A prospective survey performed in UK neurosurgical units performed from 23rd March 2020 until 24th April 2020.

Setting: Regional Neurosurgical Units outside London (as the pandemic was more advanced at time of study).

Participants: Representatives from all units were invited to collect data on new patients discussed at their MDT meetings during the study period. Each unit decided if management decision for each patient had changed due to COVID-19.

Primary and Secondary Outcome measures: Primary outcome: number of patients where the decision to undergo surgery changed compared to standard management usually offered by that MDT. Secondary outcome measures included – changes in surgical extent, numbers referred to MDT, number of patients denied surgery not receiving any treatment and reasons for any variation across the UK.

Results: 18 units (75%) provided information from 80 MDT meetings that discussed 1221 patients. 10.7% of patients had their management changed – the majority (68%) did not undergo surgery and more than half of this group not undergoing surgery had no active treatment. There was marked variation across the UK (0-28% change in management).

For peer review only - http://bmjopen.bmj.com/site/about/guidelines.xhtml

Units that did not change management could maintain capacity with dedicated oncology lists. Low volume units were less affected.

Conclusion: COVID-19 has had an impact on patients requiring surgery for malignant brain tumours, with patients receiving different treatments – most commonly not receiving surgery or any treatment at all. The variations show dedicated cancer operating lists may mitigate these pressures.

Study Registration: This study was registered with the Royal College of Surgeons of England's COVID Research Group (https://www.rcseng.ac.uk/coronavirus/rcs-covid-research-group/).

STRENGTHS AND LIMITATIONS OF THIS STUDY

- This is a National Survey that covers 75% of the UK units outside London providing a view of the impact COVID-19 had on the management of patients with brain tumours across the UK.
- Our data is based on 1221 patients.
- Data collection occurs two weeks before and two weeks after the peak of COVID-19 infections.
- We have not collected patient-level data so can't assess what happens to individual patients.
- We have only looked at surgical management and don't have data on oncology treatment.

For peer review only - http://bmjopen.bmj.com/site/about/guidelines.xhtml

BACKGROUND:

The COVID-19 pandemic has caused an unprecedented threat to healthcare delivery worldwide. Hospitals dealing with large numbers of patients requiring critical care have redeployed staff and converted operating rooms into intensive care units to cope with infected patients. This has had a marked impact on services and patient care for non-COVID-19 patients. Surgery has been disproportionately affected and requests to maintain urgent cancer surgery requires careful patient triage.[1-2]

In the UK adult brain tumours are managed across 30 regional neurosurgical centres, 24 of these are outside London. All new brain diagnoses are discussed in a multidisciplinary team meeting (MDT) that decides the optimal treatment for each patient. Each MDT will review a range of tumours from non-malignant or slow growing (low-grade) tumours whose initial treatment could be safely deferred, to the malignant tumours that are characterised by rapid growth and patient deterioration without treatment in a matter of weeks. Surgery is the main initial treatment for most patients – it can cure non-malignant tumours, remove the bulk of malignant tumours and is the only way of providing tumour tissue for pathological and molecular diagnosis to guide further treatment. Although surgery is not curative in malignant tumours it does prolong good quality survival. National guidelines recommended that surgery for malignant tumours should continue during the COVID-19 pandemic providing adjuvant oncology treatment is available.[3] The role of the MDT is critical to good patient management.

For peer review only - http://bmjopen.bmj.com/site/about/guidelines.xhtml

We undertook a national survey to evaluate the impact of COVID-19 on neurosurgical oncology services and to explore differences and variations in MDT decisions compared to the pre-COVID-19 era to investigate if patients were denied access to surgery.

For peer review only

METHODS:

Project leads from each of the UK neurosurgical units were identified to prospectively collect information from their weekly neuro-oncology MDT between 23rd March and 20th April 2020. London units were excluded as the pandemic peak was more advanced at the time of this study. Ethical approval was not required as no patient specific information was collected.

For peer review only

At each MDT meeting the following data were recorded:

- Total number of new patients discussed
- Number of patients where the MDT felt their initial/surgical treatment was different to their standard management due to COVID-19
  - Number of patients who did not receive surgery or whose surgical treatment intent was changed (e.g. biopsy rather than resection)
    - From the group that did not undergo surgery, the number of the patients who did not receive any active treatment (i.e. supportive care only).
  - Number of patients who had undergone surgery for a malignant tumour (defined as high grade glioma, metastatic tumour or other, rarer malignant tumour)

Variations in Decision Making

Data were compared to a baseline MDT workload from the 4 weeks of February (3rd – 28th February 2020) to provide information on the impact COVID-19. To explore the variations in MDT decision making, funnel plots for the proportion of cases discussed where management was altered were generated using Microsoft Excel, with 95% and 99.8%

control limits calculated using the Wilson Score method for binomially distributed variates,[4]. Project leads were invited to comment with free text on how their unit responded to COVID-19. Using this free text we developed 3 groups that arranged units based on common expressed themes of how they managed to mitigate the challenges of COVID-19.

Statistical Analysis

Statistical analysis was performed using IBM SPSS 26. All variables were assessed for normality using a 1-sample Kolmogorov–Smirnov test. Changes in weekly mean number of new patients discussed at MDT meetings and operations for malignant tumours was assessed using paired t-test. Correlation of these variables with changes of MDT decision rates was made using a Pearson correlation.

Patient Involvement

Patients/the public were not involved the concept or design of this study as this came about from concerns among professionals at the services they were providing patients. A patient/public representative was appointed to the study group during project set up to comment on the results.

For peer review only

RESULTS:

18 of the 24 eligible neurosurgical units (75%) participated in the study. 1221 new patients were included from 80 MDT meetings. Comparing activity to baseline, there was a significant reduction in the number of new patients discussed from a mean weekly baseline (Table 1). 131 patients (10.7%) had a change in their initial management due to COVID-19. Of these, 15 (11.5%) had a change of surgical intent and 90 (68%) had no surgery at all. 47 patients (52% of those decided for no surgery) were decided for best supportive care.

The change in initial management varied between centres and ranged from 0 to 28%. This did not correlate with either the change in number of weekly operations (R=-0.03; P=0.91) nor number of patients discussed at an MDT meeting (R=-0.29; P=0.29). The funnel plot identified three groups of units (Figure 1). Group A was smaller units with low numbers of new patients (mean = 7.4, 95%CI [4.1 – 10.6]) and low rates of management changes. They were either less affected by COVID or were able to cope with the numbers of referrals. The group included a unit whose numbers decreased from 28.5 to 13.6 per week (52% reduction). Group B was larger units (mean weekly new patients = 34.1, 95% CI [20.9, 47.2]) that allocated dedicated oncology lists. This was achieved by a variety of methods – implementing daily oncology lists, managing patients as emergencies or using the private hospital sector. Group C was the group with the largest change in management. A common theme for this group was the lack of dedicated oncology lists – patients with malignant tumours competing for surgical theatre operating time with other urgent surgical cases.

For peer review only - http://bmjopen.bmj.com/site/about/guidelines.xhtml

Patient/public interpretation of results.

The results were examined by the Study Group's patient/public representative (HB). They commented that "*when you're diagnosed with a brain tumour, your whole life is not only upended but put on hold. At this point, patients and their families need certainty. To be denied surgery - the first line treatment - doesn't bear thinking about. This impacts on the whole of the patient pathway, as without biopsies and resections, nobody really knows what you are treating, or how much tumour you are treating. It undermines the whole of the personalised medicine agenda and this cohort of patients will be living with the consequences, but probably not for as long as they would have been if they had had surgery. The short-sighted decision to bypass brain tumour surgery is undoubtedly shortening patients' lives.*"

For peer review only

DISCUSSION

This national study has shown the impact COVID-19 has had on MDT decision making for malignant (gliomas and metastases) brain tumour patients. 10.7% of patients had their initial management changed, of which the majority (68%) did not undergo surgery. Over half of these patients had palliative care only. Surgery is the first line treatment in malignant brain tumours and extent of resection is the only modifiable prognostic factor.[5] Patients with glioblastomas, the commonest malignant primary tumour, have a very limited survival without treatment,[6] and surgical resection improves efficacy of adjuvant therapies and quality of life.[7]

Like other cancers, COVID-19 has impacted on referrals.[8] We found a 27% reduction in a number of patients discussed and operations performed for malignant tumours (26% reduction) (cf. Table 1). Initial symptoms of brain tumours are frequently non-specific and patients often present when they decompensate.[9-10] The reduction in new tumour referrals during this study may result in patients presenting later and with more extensive disease.

An interesting finding was the variation between units. Group analysis identified trends in units that successfully reduced the impact of COVID-19 on MDT management. Strategies to ensure that dedicated cancer lists continued were helpful in larger units. It is clear that where cancer patients were scheduled on operating lists with other urgent cases more substantial MDT management changes were needed to cope.

For peer review only - http://bmjopen.bmj.com/site/about/guidelines.xhtml

The study does have limitations. Although this survey covered 70% of units, not all geographical areas have been included (e.g. South West and London). We have not been able to include the COVID-19 infection rate across the country into our analysis since each neurosurgery unit serves a large geographical area. Our study only analysed changes in surgical treatment, but we know there are also changes in adjuvant therapy, such as radiation dose and chemotherapy regimes. It was difficult to quantify these changes in our study since patients are often treated in oncology centres that are separate from the neurosurgery centres. The main focus of our study was to look at the MDT decision making where decisions about adjuvant therapy were made to influence surgery as national guidance recommend surgery for patients with malignant tumours only where adjuvant therapy was available.

In conclusion, COVID-19 has had an impact on patients requiring surgery for malignant brain tumours – with 10.7% having a change of management due to COVID-19, most commonly by not having surgery and many having no active treatment at all. The variation of changes in decision making show that dedicated cancer operating lists may help to mitigate the pressures of COVID-19.

For peer review only - http://bmjopen.bmj.com/site/about/guidelines.xhtml

Additional Information:

• Acknowledgements

Members of the COVID-CNSMDT Study group that delivered this study are (* project lead)

Belfast: Thomas Flannery* and Nicola Johnson

Birmingham: Victoria Wykes*, Georgios Solomou, Yasir Chowdhury and Colin Watts

Brighton: Giles Critchley* and Mandy Lynch

Bristol: Neil Barua*, George Malcolm, Ven Iyer, Constantinos Charalambides and Chris Herbert

Cambridge: Stephen Price*, Alexis Joannides, Richard Mair, Thomas Santarius , Fiona Harris and Olivia Poulter

Coventry: Fardad Afshari*, Georgios Solomou and Sandeep Solanki

Dundee: Anna Solth* and Kismet Hossain-Ibrahim

Edinburgh: Michael Poon* and Paul Brennan

Hull: Chittoor Rajaraman *, Shailendra Achawal and Gerry O'Reilly

Leeds: Ryan Mathew*, John Goodden and Simon Thomson

Liverpool: Michael D Jenkinson*, Andrew R Brodbelt, Emmanuel Chavredakis, David DA Lawson and Jibril Farah

Manchester: Tina Karabatsou* and Daniel Fountain.

Newcastle: Surash Surash*, Syed Shumon and Damian Holliman

Nottingham: Stuart Smith*, Muhammed Zafar, William Sage and Murugan Sitaraman

Oxford: Rory Piper* and Puneet Plaha

Sheffield: Ola Rominiyi* and Yahia Al-Tamimi

Southampton: Huan Wee Chan* and Paul Grundy

For peer review only - http://bmjopen.bmj.com/site/about/guidelines.xhtml

Stoke-on-Trent: Erminia Albanese* and Ebere Ogbonnaya

• Authors' contributions

SJP, MDJ, PP and *PJH* were involved in the study concept and design.

SJP, TF, VW, GC, NB, FA, AS, MP, RC, RM, MDJ, DF, SSu, SSm, GS, RP, OR, HWC and *EA* were involved in collecting data from each site and guarantee the data at each site.

SJP and AJ were involved in data analysis.

HB provided the patient perspective on our findings.

SJP wrote the first draft of the paper and *all authors* were involved in revising the paper and have approved the final paper.

• Ethics approval and consent to participate

As this study did not collect personal data ethical approval was not required. It was registered as a service evaluation in each site.

• Consent for publication

No specific consent is required for publication.

• Data availability

Data access queries should be addressed to the corresponding author (SJP). All data is anonymised and individual unit level data is not available. Approval for data release will be made by SJP, MDJ, PP and PJH who conceived the study.

For peer review only - http://bmjopen.bmj.com/site/about/guidelines.xhtml

• Conflict of interest

None of the authors have conflicts of interest.

• Funding

SJP is funded by a National Institute for Health Research (NIHR), (Career Development Fellowship).

This paper presents independent research funded by the National Institute for Health Research (NIHR). The views expressed are those of the author(s) and not necessarily those of the NHS, the NIHR or the Department of Health and Social Care.

For peer review only

REFERENCES

1. Al-Jabir A, Kerwan A, Nicola M, et al. Impact of the coronavirus (COVID-19) pandemic on surgical practice - Part 2 (surgical prioritisation). *Int J Sur* 2020 May 12, 10.1016/j.ijsu.2020.05.002.

2. Stevens S, Pritchard A. Important and urgent – next steps on NHS response to COVID-19. https://www.england.nhs.uk/coronavirus/wp-content/uploads/sites/52/2020/03/urgent-next-steps-on-nhs-response-to-covid-19-letter-simon-stevens.pdf (accessed March 2020).

3. The Society of British Neurological Surgeons. Adult Neuro-Oncology service provision during COVID-19 outbreak. https://www.sbns.org.uk/index.php/download_file /view/1646/1224/416/1224/. (accessed March 2020).

4. Spiegelhalter DJ. Funnel plots for comparing institutional performance. *Statistics in medicine* 2005;24(8):1185-1202.

5. Brown TJ, Brennan MC, Li M, et al. Association of the extent of resection with survival in glioblastoma: a systematic review and meta-analysis. *JAMA Oncol.* 2016;2(11):1460-1469.

6. Brodbelt A, Greenberg D, Winters T, et al. Glioblastoma in England: 2007-2011. *Eur J Cancer* 2015;51(4):533-542.

For peer review only - http://bmjopen.bmj.com/site/about/guidelines.xhtml

7. Stummer W, van den Bent MJ, Westphal M. Cytoreductive surgery of glioblastoma as the key to successful adjuvant therapies: new arguments in an old discussion. *Acta Neurochir (Wien)* 2011;153(6):1211-1218.

8. Dinmohamed AG, Visser O, Verhoeven RHA, et al. Fewer cancer diagnoses during the COVID-19 epidemic in the Netherlands. *The Lancet Oncology* 2020 Apr 30. doi 10.1016/s1470-2045(20)30265-5.

9. Walter FM, Penfold C, Joannides A, et al. Missed opportunities for diagnosing brain tumours in primary care: a qualitative study of patient experiences. *Br J Gen Pract* 2019;69(681):e224-e235.

10. Abel GA, Shelton J, Johnson S, Elliss-Brookes L, et al. Cancer-specific variation in emergency presentation by sex, age and deprivation across 27 common and rarer cancers. *Br J Cancer* 2015;112(Suppl 1):S129-136.

For peer review only

For peer review only - http://bmjopen.bmj.com/site/about/guidelines.xhtml

TABLE AND FIGURE LEGENDS

Table 1: Changes in workload between baseline and COVID-19 period. Malignant tumours refer to high grade gliomas, metastases and other rarer malignant tumours.

Figure 1: Funnel plot showing variation in number of patients whose management was changed due to COVID-19 vs. the number of patients discussed in MDTs over the study period. Standard deviation curves have been included. We have broken the data into 3 groups with different responses to the pandemic.

For peer review only

For peer review only - http://bmjopen.bmj.com/site/about/guidelines.xhtml

TABLE 1

	Baseline	During study period	Significance	Difference
**Number of new patients**	21.2 [95%CI 14.0 – 28.3]	15.3 [95%CI 10.0 – 20.6]	t=-4.26, P=0.0005	↓27%
**Number of operations for malignant tumours**	4.1 [95%CI 3.2 – 5.0]	3.0 [95%CI 2.0 – 4.1]	t=-2.76, P=0.01	↓26%

For peer review only

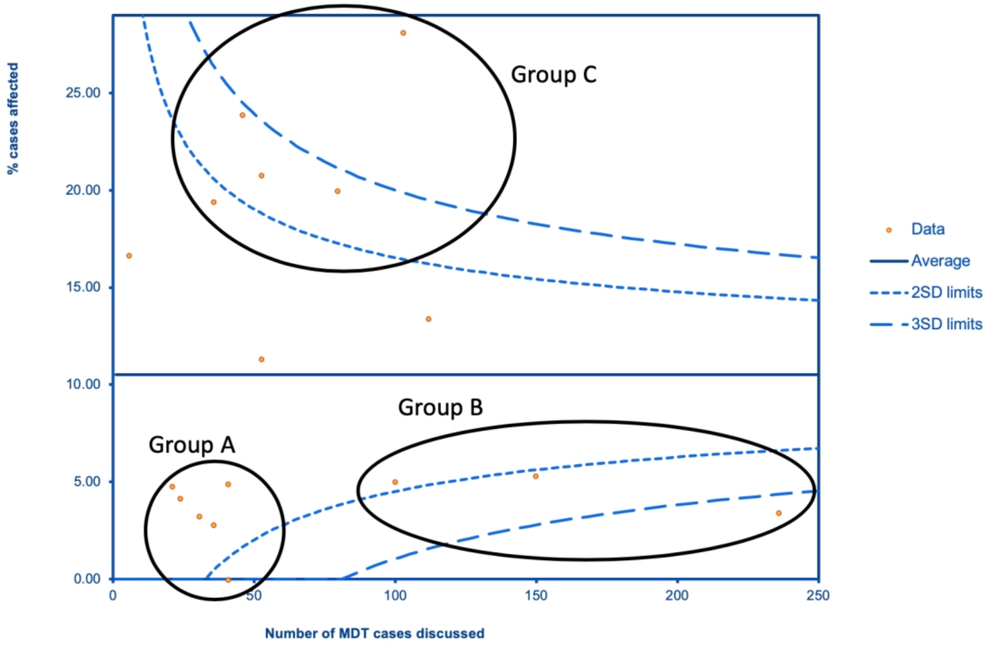

Figure 1: Funnel plot showing variation in number of patients whose management was changed due to COVID-19 vs. the number of patients discussed in MDTs over the study period. Standard deviation curves have been included. We have broken the data into 3 groups with different responses to the pandemic.

612x402mm (72 x 72 DPI)

BMJ Open

Impact of COVID-19 Pandemic on Surgical Neuro-oncology Multi-Disciplinary Team Decision Making – A National Survey (COVID-CNSMDT Study)

Journal:	*BMJ Open*
Manuscript ID	bmjopen-2020-040898.R1
Article Type:	Original research
Date Submitted by the Author:	03-Jul-2020
Complete List of Authors:	Price, Stephen; Cambridge University, Neurosurgery Division, Dept. Clinical Neurosciences<br>Joannides, Alexis; Cambridge University, Neurosurgery Division, Department of Clinical Neurosciences<br>Plaha, Puneet; John Radcliffe Hospital, Department of Neurosurgery<br>Afshari, Fardad; University Hospitals Coventry and Warwickshire NHS Trust, Department of Neurosurgery<br>Albanese, Erminia; University Hospitals of North Midlands NHS Trust, Department of Neurosurgery<br>Barua, Neil; North Bristol NHS Trust, Department of Neurosurgery<br>Chan, Huan Wee; University Hospital Southampton NHS Foundation Trust, Department of Neurosurgery<br>Critchley, Giles; Brighton and Sussex University Hospitals NHS Trust, Department of Neurosurgery<br>Flannery, Thomas; Royal Victoria Hospital<br>Fountain, Daniel; Salford Royal NHS Foundation Trust, Manchester Centre for Clinical Neurosciences<br>Mathew, Ryan; Leeds General Infirmary, Department of Neurosurgery<br>Piper, Rory; John Radcliffe Hospital, Department of Neurosurgery<br>Poon, Michael; The University of Edinburgh, Usher Institute<br>Rajaraman, Chittoor; Hull Royal Infirmary, Department of Neurosurgery<br>Rominiyi, Ola; Royal Hallamshire Hospital, Department of Neurosurgery<br>Smith, Stuart; Queen's Medical Centre, Department of Neurosurgery<br>Solomou, Georgios; University Hospitals Coventry and Warwickshire NHS Trust, Department of Neurosurgery; University of Birmingham College of Medical and Dental Sciences<br>Solth, Anna; Ninewells Hospital, Department of Neurosurgery<br>Surash, Surash; Royal Victoria Infirmary, Department of Neurosurgery<br>Wykes, Victoria; Queen Elizabeth Hospital, Department of Neurosurgery<br>Watts, Colin; University of Birmingham, Institute of Cancer and Genomic Studies<br>Bulbeck, Helen; brainstrust<br>Hutchinson, Peter; Cambridge University, Academic Neurosurgery Division, Department of Clinical Neurosciences<br>Jenkinson, Michael; The Walton Centre NHS Foundation Trust, Department of Neurosurgery
**Primary Subject Heading**:	Oncology

Secondary Subject Heading:	Neurology, Surgery
Keywords:	Neurological oncology < ONCOLOGY, Change management < HEALTH SERVICES ADMINISTRATION & MANAGEMENT, INFECTIOUS DISEASES

SCHOLARONE™
Manuscripts

I, the Submitting Author has the right to grant and does grant on behalf of all authors of the Work (as defined in the below author licence), an exclusive licence and/or a non-exclusive licence for contributions from authors who are: i) UK Crown employees; ii) where BMJ has agreed a CC-BY licence shall apply, and/or iii) in accordance with the terms applicable for US Federal Government officers or employees acting as part of their official duties; on a worldwide, perpetual, irrevocable, royalty-free basis to BMJ Publishing Group Ltd ("BMJ") its licensees and where the relevant Journal is co-owned by BMJ to the co-owners of the Journal, to publish the Work in this journal and any other BMJ products and to exploit all rights, as set out in our licence.

The Submitting Author accepts and understands that any supply made under these terms is made by BMJ to the Submitting Author unless you are acting as an employee on behalf of your employer or a postgraduate student of an affiliated institution which is paying any applicable article publishing charge ("APC") for Open Access articles. Where the Submitting Author wishes to make the Work available on an Open Access basis (and intends to pay the relevant APC), the terms of reuse of such Open Access shall be governed by a Creative Commons licence – details of these licences and which Creative Commons licence will apply to this Work are set out in our licence referred to above.

Other than as permitted in any relevant BMJ Author's Self Archiving Policies, I confirm this Work has not been accepted for publication elsewhere, is not being considered for publication elsewhere and does not duplicate material already published. I confirm all authors consent to publication of this Work and authorise the granting of this licence.

For peer review only

Impact of COVID-19 Pandemic on Surgical Neuro-oncology Multi-Disciplinary Team Decision Making – A National Survey (COVID-CNSMDT Study)

Stephen J Price[1], Alexis J Joannides[1], Puneet Plaha[2], Fardad T Afshari[3], Erminia Albanese[4], Neil U Barua[5], Huan Wee Chan[6], Giles Critchley[7], Thomas Flannery[8], Daniel M Fountain[9], Ryan Mathew[10], Rory J Piper[2], Michael TC Poon[11], Chittoor Rajaraman[12], Ola Rominiyi[13], Stuart Smith[14], Georgios Solomou[3,15], Anna Solth[16], Surash Surash[17], Victoria Wykes[18], Colin Watts[18], Helen Bulbeck[19], Peter Hutchinson[1] and Michael D Jenkinson[20]

On behalf of the COVID-CNSMDT study group

[1]Neurosurgery Division, Dept. Clinical Neurosciences, University of Cambridge, Cambridge CB2 0QQ; [2] Dept of Neurosurgery, John Radcliffe Hospital, Oxford OX3 9DU; [3] Dept of Neurosurgery, University Hospital Coventry, CV2 2DX; [4]Dept. of Neurosurgery, University Hospital of North Midlands, Stoke on Trent, ST46QG; [5]Dept. of Neurosurgery, North Bristol NHS Trust, Bristol BS10 5NB; [6]Dept of Neurosurgery, Wessex Neurological Centre, Southampton SO16 6YD; [7]Dept of Neurosurgery, Brighton and Sussex University Hospitals NHS Trust, Brighton BN2 5BE; [8]Dept of Neurosurgery, Royal Victoria Hospital, Belfast BT12 6BA; [9] Manchester Centre for Clinical Neurosciences, Salford Royal NHS Foundation Trust, Salford M6 8HD, [10]Dept of Neurosurgery, General Infirmary at Leeds, Leeds LS1 3EX; [11]Usher Institute, University of Edinburgh, EH16 4SB; [12]Dept of Neurosurgery, Hull Royal Infirmary, Hull HU3 2JZ; [13]Dept of Neurosurgery, Royal Hallamshire Hospital, Sheffield S10 2JF; [14]Dept of Neurosurgery, Queen's Medical Centre, Nottingham NG7 2UH; [15]University of Birmingham, Birmingham B15 2TT; [16]Dept of Neurosurgery, Ninewells Hospital and Medical

For peer review only - http://bmjopen.bmj.com/site/about/guidelines.xhtml

School, Dundee DD1 9SY; [17]Dept of Neurosurgery, Royal Victoria Infirmary, Newcastle upon Tyne NE1 4L; [18]Dept of Neurosurgery, Queen Elizabeth Neuroscience Centre, Birmingham B15 2TB; [19]Brainstrust, Cowes, Isle of Wight PO31 7QG; [20]Dept of Neurosurgery, University of Liverpool & The Walton Centre NHS Trust, Liverpool, L9 7LJ.

Corresponding Author:

Mr Stephen J Price

Hon. Consultant Neurosurgeon

Department of Clinical Neurosciences,

Division of Neurosurgery,

Box 167 Cambridge Biomedical Campus,

Cambridge CB2 0QQ.

E-mail: sjp58@cam.ac.uk

Tel: 01223 746455

WORD COUNT: 1741 words

For peer review only

For peer review only - http://bmjopen.bmj.com/site/about/guidelines.xhtml

ABSTRACT:

Objectives: Pressures on healthcare systems due to COVID-19 has impacted non-COVID patients with surgery disproportionally affected. This study aims to understand the impact on the initial management of patients with brain tumours by measuring changes to normal multidisciplinary team (MDT) decision making.

Design: A prospective survey performed in UK neurosurgical units performed from 23rd March 2020 until 24th April 2020.

Setting: Regional Neurosurgical Units outside London (as the pandemic was more advanced at time of study).

Participants: Representatives from all units were invited to collect data on new patients discussed at their MDT meetings during the study period. Each unit decided if management decision for each patient had changed due to COVID-19.

Primary and Secondary Outcome measures: Primary outcome: number of patients where the decision to undergo surgery changed compared to standard management usually offered by that MDT. Secondary outcome measures included – changes in surgical extent, numbers referred to MDT, number of patients denied surgery not receiving any treatment and reasons for any variation across the UK.

Results: 18 units (75%) provided information from 80 MDT meetings that discussed 1221 patients. 10.7% of patients had their management changed – the majority (68%) did not undergo surgery and more than half of this group not undergoing surgery had no active treatment. There was marked variation across the UK (0-28% change in management).

For peer review only - http://bmjopen.bmj.com/site/about/guidelines.xhtml

Units that did not change management could maintain capacity with dedicated oncology lists. Low volume units were less affected.

Conclusion: COVID-19 has had an impact on patients requiring surgery for malignant brain tumours, with patients receiving different treatments – most commonly not receiving surgery or any treatment at all. The variations show dedicated cancer operating lists may mitigate these pressures.

Study Registration: This study was registered with the Royal College of Surgeons of England's COVID Research Group (https://www.rcseng.ac.uk/coronavirus/rcs-covid-research-group/).

For peer review only

STRENGTHS AND LIMITATIONS OF THIS STUDY

- This is a National Survey that covers 75% of the UK units outside London providing a view of the impact COVID-19 had on the management of patients with brain tumours across the UK.
- Our data is based on 1221 patients.
- Data collection occurs two weeks before and two weeks after the peak of COVID-19 infections.
- We have not collected patient-level data so can't assess what happens to individual patients.
- We have only looked at surgical management and don't have data on oncology treatment.

For peer review only - http://bmjopen.bmj.com/site/about/guidelines.xhtml

BACKGROUND:

The COVID-19 pandemic has caused an unprecedented threat to healthcare delivery worldwide. Hospitals dealing with large numbers of patients requiring critical care have redeployed staff and converted operating rooms into intensive care units to cope with infected patients. This has had a marked impact on services and patient care for non-COVID-19 patients. Surgery has been disproportionately affected and requests to maintain urgent cancer surgery requires careful patient triage.[1-2]

In the UK adult brain tumours are managed across 30 regional neurosurgical centres, 24 of these are outside London. All new brain diagnoses are discussed in a multidisciplinary team meeting (MDT) that decides the optimal treatment for each patient. Each MDT will review a range of tumours from non-malignant or slow growing (low-grade) tumours whose initial treatment could be safely deferred, to the malignant tumours that are characterised by rapid growth and patient deterioration without treatment in a matter of weeks. Surgery is the main initial treatment for most patients – it can cure non-malignant tumours, remove the bulk of malignant tumours and is the only way of providing tumour tissue for pathological and molecular diagnosis to guide further treatment. Although surgery is not curative in malignant tumours it does prolong good quality survival. National guidelines recommended that surgery for malignant tumours should continue during the COVID-19 pandemic providing adjuvant oncology treatment is available.[3] The role of the MDT is critical to good patient management.

For peer review only - http://bmjopen.bmj.com/site/about/guidelines.xhtml

We undertook a national survey to evaluate the impact of COVID-19 on neurosurgical oncology services and to explore differences and variations in MDT decisions compared to the pre-COVID-19 era to investigate if patients were denied access to surgery.

For peer review only

For peer review only - http://bmjopen.bmj.com/site/about/guidelines.xhtml

METHODS:

Project leads from each of the UK neurosurgical units were identified to prospectively collect information from their weekly neuro-oncology MDT between 23rd March and 20th April 2020. London units were excluded as the pandemic peak was more advanced at the time of this study. Ethical approval was not required as no patient specific information was collected.

The primary outcome measure in this study is the change in MDT decision making. This was determined by each MDT. At each MDT meeting the project lead would review the decisions made for every patient with the rest of the team and decide whether this decision had differed from their normal practice due to COVID.

At each MDT meeting the following data were recorded:

- Total number of new patients discussed
- Number of patients where the MDT felt their initial/surgical treatment was different to their standard management due to COVID-19
  - Number of patients who did not receive surgery or whose surgical treatment intent was changed (e.g. biopsy rather than resection)
    - From the group that did not undergo surgery, the number of the patients who did not receive any active treatment (i.e. supportive care only).
  - Number of patients who had undergone surgery for a malignant tumour (defined as high grade glioma, metastatic tumour or other, rarer malignant tumour)

For peer review only - http://bmjopen.bmj.com/site/about/guidelines.xhtml

Variations in Decision Making

Data were compared to a baseline MDT workload from the 4 weeks of February (3rd – 28th February 2020) to provide information on the impact COVID-19. To explore the variations in MDT decision making, funnel plots for the proportion of cases discussed where management was altered were generated using Microsoft Excel, with 95% and 99.8% control limits calculated using the Wilson Score method for binomially distributed variates,[4]. Project leads were invited to comment with free text on how their unit responded to COVID-19. Using this free text we developed 3 groups that arranged units based on common expressed themes of how they managed to mitigate the challenges of COVID-19.

Statistical Analysis

Statistical analysis was performed using IBM SPSS 26. All variables were assessed for normality using a 1-sample Kolmogorov–Smirnov test. Changes in weekly mean number of new patients discussed at MDT meetings and operations for malignant tumours was assessed using paired t-test. Correlation of these variables with changes of MDT decision rates was made using a Pearson correlation.

Patient Involvement

Patients/the public were not involved the concept or design of this study as this came about from concerns among professionals at the services they were providing patients. A

For peer review only - http://bmjopen.bmj.com/site/about/guidelines.xhtml

patient/public representative was appointed to the study group during project set up to comment on the results.

For peer review only - http://bmjopen.bmj.com/site/about/guidelines.xhtml

RESULTS:

18 of the 24 eligible neurosurgical units (75%) participated in the study. 1221 new patients were included from 80 MDT meetings. In all units the functioning of the MDT changed with 8 units (33%) moving to fully video conferencing, all of the other units would limit attendance to a smaller number of senior representatives of different specialities (e.g. neurosurgery, oncology, radiology etc). 4 units (17%) reduced the duration of the meeting.

Comparing activity to baseline, there was a significant reduction in the number of new patients discussed from a mean weekly baseline (Table 1). 131 patients (10.7%) had a change in their initial management due to COVID-19. Of these, 15 (11.5%) had a change of surgical intent and 90 (68%) had no surgery at all. 47 patients (52% of those decided for no surgery) were decided for best supportive care. Information from project leads suggest the majority of these patients were elderly and had poor performance status. Other reasons for not operating were patients with low-grade gliomas or meningiomas whose surgery was deferred until after the critical phase of the pandemic.

The change in initial management varied between centres and ranged from 0 to 28%. This did not correlate with either the change in number of weekly operations (R=-0.03; P=0.91) nor number of patients discussed at an MDT meeting (R=-0.29; P=0.29). The funnel plot identified three groups of units (Figure 1). Group A was smaller units with low numbers of new brain tumour patients (mean = 7.4, 95%CI [4.1 – 10.6]) and low rates of management changes. Project leads commented that COVID had less impact on their units or were able to cope with the numbers of referrals. The group included a unit whose numbers decreased

For peer review only - http://bmjopen.bmj.com/site/about/guidelines.xhtml

from 28.5 to 13.6 per week (52% reduction). Group B was larger units (mean weekly new patients = 34.1, 95% CI [20.9, 47.2]) that allocated dedicated oncology lists. This was achieved by a variety of methods – implementing daily oncology lists, managing patients as emergencies or using the private hospital sector to provide space to treat brain tumour patients. Group C was the group with the largest change in management. A common theme for this group was the lack of dedicated oncology lists – patients with malignant tumours competing for surgical theatre operating time with other urgent surgical cases.

Patient/public interpretation of results.

The results were examined by the Study Group's patient/public representative (HB). They commented that "*when you're diagnosed with a brain tumour, your whole life is not only upended but put on hold. At this point, patients and their families need certainty. To be denied surgery - the first line treatment - doesn't bear thinking about. This impacts on the whole of the patient pathway, as without biopsies and resections, nobody really knows what you are treating, or how much tumour you are treating. It undermines the whole of the personalised medicine agenda and this cohort of patients will be living with the consequences, but probably not for as long as they would have been if they had had surgery. The short-sighted decision to bypass brain tumour surgery is undoubtedly shortening patients' lives.*"

For peer review only - http://bmjopen.bmj.com/site/about/guidelines.xhtml

DISCUSSION

This national study has shown the impact COVID-19 has had on MDT decision making for malignant (gliomas and metastases) brain tumour patients. 10.7% of patients had their initial management changed, of which the majority (68%) did not undergo surgery. Over half of these patients had palliative care only. Surgery is the first line treatment in malignant brain tumours and extent of resection is the only modifiable prognostic factor.[5] Patients with glioblastomas, the commonest malignant primary tumour, have a very limited survival without treatment,[6] and surgical resection improves efficacy of adjuvant therapies and quality of life.[7]

Like other cancers, COVID-19 has impacted on referrals.[8] We found a 27% reduction in a number of patients discussed and operations performed for malignant tumours (26% reduction) (cf. Table 1). Initial symptoms of brain tumours are frequently non-specific and patients often present when they decompensate.[9-10] The reduction in new tumour referrals during this study may result in patients presenting later and with more extensive disease.

An interesting finding was the variation between units. Group analysis identified trends in units that successfully reduced the impact of COVID-19 on MDT management. Strategies to ensure that dedicated cancer lists continued were helpful in larger units. It is clear that where cancer patients were scheduled on operating lists with other urgent cases more substantial MDT management changes were needed to cope. Our findings complement

For peer review only - http://bmjopen.bmj.com/site/about/guidelines.xhtml

other studies that have shown that the use of the private sector during this COVID pandemic results in less disruption to services.[11]

The study does have limitations. Although this survey covered 70% of units, not all geographical areas have been included (e.g. South West and London). We have not been able to include the COVID-19 infection rate across the country into our analysis since each neurosurgery unit serves a large geographical area. The dates of our survey, however, correspond to the peak in cases for every geographical region.

Our primary outcome measure was, by its nature, pragmatic. This introduces some subjectivity between units as to what they defined as a change in management. As most of these changes involved no surgery or a change in surgical intent, we believe project leads reported significant changes in management that this study sought to explore. Our study only analysed changes in surgical treatment, but we know there are also changes in adjuvant therapy, such as radiation dose and chemotherapy regimes. It was difficult to quantify these changes in our study since patients are often treated in oncology centres that are separate from the neurosurgery centres. The main focus of our study was to look at the MDT decision making where decisions about adjuvant therapy were made to influence surgery as national guidance recommend surgery for patients with malignant tumours only where adjuvant therapy was available.

In conclusion, COVID-19 has had an impact on patients requiring surgery for malignant brain tumours – with 10.7% having a change of management due to COVID-19, most commonly

For peer review only - http://bmjopen.bmj.com/site/about/guidelines.xhtml

by not having surgery and many having no active treatment at all. The variation of changes in decision making show that dedicated cancer operating lists may help to mitigate the pressures of COVID-19.

Additional Information:

• **Acknowledgements**

Members of the COVID-CNSMDT Study group that delivered this study are (* project lead)

Belfast: Thomas Flannery* and Nicola Johnson

Birmingham: Victoria Wykes*, Georgios Solomou, Yasir Chowdhury and Colin Watts

Brighton: Giles Critchley* and Mandy Lynch

Bristol: Neil Barua*, George Malcolm, Ven Iyer, Constantinos Charalambides and Chris Herbert

Cambridge: Stephen Price*, Alexis Joannides, Richard Mair, Thomas Santarius , Fiona Harris and Olivia Poulter

Coventry: Fardad Afshari*, Georgios Solomou and Sandeep Solanki

Dundee: Anna Solth* and Kismet Hossain-Ibrahim

Edinburgh: Michael Poon* and Paul Brennan

Hull: Chittoor Rajaraman *, Shailendra Achawal and Gerry O'Reilly

Leeds: Ryan Mathew*, John Goodden and Simon Thomson

Liverpool: Michael D Jenkinson*, Andrew R Brodbelt, Emmanuel Chavredakis, David DA Lawson and Jibril Farah

Manchester: Tina Karabatsou* and Daniel Fountain.

Newcastle: Surash Surash*, Syed Shumon and Damian Holliman

For peer review only
For peer review only - http://bmjopen.bmj.com/site/about/guidelines.xhtml

Nottingham: Stuart Smith*, Muhammed Zafar, William Sage and Murugan Sitaraman

Oxford: Rory Piper* and Puneet Plaha

Sheffield: Ola Rominiyi* and Yahia Al-Tamimi

Southampton: Huan Wee Chan* and Paul Grundy

Stoke-on-Trent: Erminia Albanese* and Ebere Ogbonnaya

• Authors' contributions

SJP, MDJ, PP, CW and *PJH* were involved in the study concept and design.

SJP, TF, VW, GC, NB, FA, AS, MP, RC, RM, MDJ, DF, SSu, SSm, GS, RP, OR, HWC and *EA* were involved in collecting data from each site and guarantee the data at each site.

SJP and AJ were involved in data analysis.

HB provided the patient perspective on our findings.

SJP wrote the first draft of the paper and *all authors* were involved in revising the paper and have approved the final paper.

• Ethics approval and consent to participate

As this study did not collect personal data ethical approval was not required. It was registered as a service evaluation in each site.

• Consent for publication

No specific consent is required for publication.

• Data availability

For peer review only - http://bmjopen.bmj.com/site/about/guidelines.xhtml

Data access queries should be addressed to the corresponding author (SJP). All data is anonymised and individual unit level data is not available. Approval for data release will be made by SJP, MDJ, PP, CW and PJH who conceived the study.

• Conflict of interest

None of the authors have conflicts of interest.

• Funding

SJP is funded by a National Institute for Health Research (NIHR), (Career Development Fellowship). This paper presents independent research funded by the National Institute for Health Research (NIHR). The views expressed are those of the author(s) and not necessarily those of the NHS, the NIHR or the Department of Health and Social Care.

For peer review only

For peer review only - http://bmjopen.bmj.com/site/about/guidelines.xhtml

REFERENCES

1. Al-Jabir A, Kerwan A, Nicola M, et al. Impact of the coronavirus (COVID-19) pandemic on surgical practice - Part 2 (surgical prioritisation). *Int J Sur* 2020 May 12, 10.1016/j.ijsu.2020.05.002.

2. Stevens S, Pritchard A. Important and urgent – next steps on NHS response to COVID-19. https://www.england.nhs.uk/coronavirus/wp-content/uploads/sites/52/2020/03/urgent-next-steps-on-nhs-response-to-covid-19-letter-simon-stevens.pdf (accessed March 2020).

3. The Society of British Neurological Surgeons. Adult Neuro-Oncology service provision during COVID-19 outbreak. https://www.sbns.org.uk/index.php/download_file /view/1646/1224/416/1224/. (accessed March 2020).

4. Spiegelhalter DJ. Funnel plots for comparing institutional performance. *Statistics in medicine* 2005;24(8):1185-1202.

5. Brown TJ, Brennan MC, Li M, et al. Association of the extent of resection with survival in glioblastoma: a systematic review and meta-analysis. *JAMA Oncol.* 2016;2(11):1460-1469.

6. Brodbelt A, Greenberg D, Winters T, et al. Glioblastoma in England: 2007-2011. *Eur J Cancer* 2015;51(4):533-542.

For peer review only - http://bmjopen.bmj.com/site/about/guidelines.xhtml

7. Stummer W, van den Bent MJ, Westphal M. Cytoreductive surgery of glioblastoma as the key to successful adjuvant therapies: new arguments in an old discussion. *Acta Neurochir (Wien)* 2011;153(6):1211-1218.

8. Dinmohamed AG, Visser O, Verhoeven RHA, et al. Fewer cancer diagnoses during the COVID-19 epidemic in the Netherlands. *The Lancet Oncology* 2020 Apr 30. doi 10.1016/s1470-2045(20)30265-5.

9. Walter FM, Penfold C, Joannides A, et al. Missed opportunities for diagnosing brain tumours in primary care: a qualitative study of patient experiences. *Br J Gen Pract* 2019;69(681):e224-e235.

10. Abel GA, Shelton J, Johnson S, Elliss-Brookes L, et al. Cancer-specific variation in emergency presentation by sex, age and deprivation across 27 common and rarer cancers. *Br J Cancer* 2015;112(Suppl 1):S129-136.

11. Jean WC, Ironside NT, Sack KD, et al. The impact of COVID-19 on neurosurgeons and the strategy for triaging non-emergent operations: a global neurosurgery study. *Acta Neurochir (Wien)* 2020;162(6):1229-40.

For peer review only

TABLE AND FIGURE LEGENDS

Table 1: Changes in workload between baseline and COVID-19 period. Malignant tumours refer to high grade gliomas, metastases and other rarer malignant tumours.

Figure 1: Funnel plot showing variation in number of patients whose management was changed due to COVID-19 vs. the number of patients discussed in MDTs over the study period. Standard deviation curves have been included. We have broken the data into 3 groups with different responses to the pandemic.

For peer review only

For peer review only - http://bmjopen.bmj.com/site/about/guidelines.xhtml

TABLE 1

	Baseline	During study period	Significance	Difference
**Number of new patients**	21.2 [95%CI 14.0 – 28.3]	15.3 [95%CI 10.0 – 20.6]	t=-4.26, P=0.0005	↓27%
**Number of operations for malignant tumours**	4.1 [95%CI 3.2 – 5.0]	3.0 [95%CI 2.0 – 4.1]	t=-2.76, P=0.01	↓26%

For peer review only

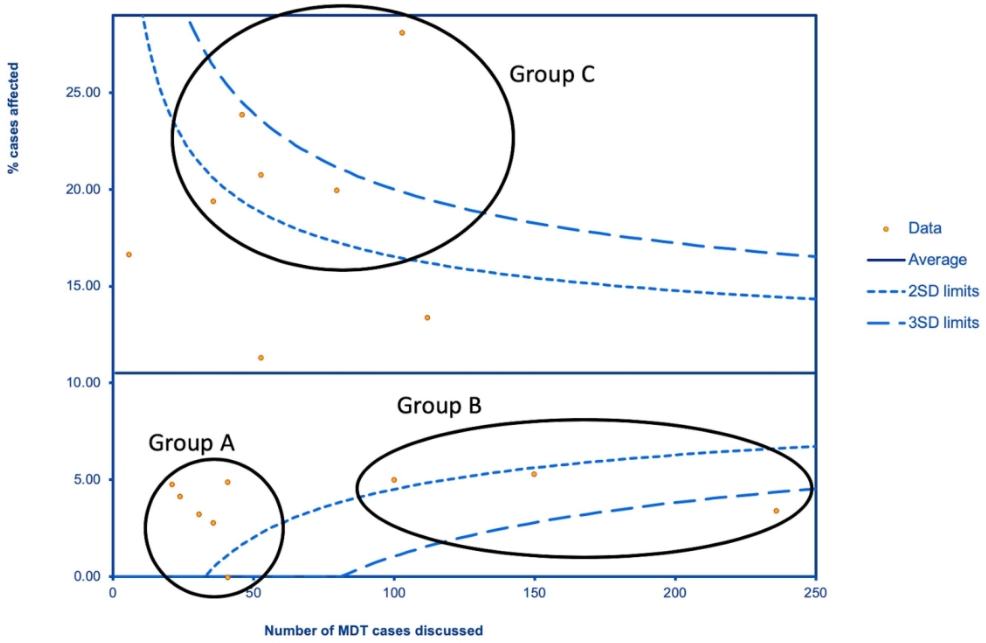

FIGURE 1: Funnel plot showing variation in number of patients whose management was changed due to COVID-19 vs. the number of patients discussed in MDTs over the study period. Standard deviation curves have been included. We have broken the data into 3 groups with different responses to the pandemic.

137x90mm (300 x 300 DPI)

BMJ Open

Impact of COVID-19 Pandemic on Surgical Neuro-oncology Multi-Disciplinary Team Decision Making – A National Survey (COVID-CNSMDT Study)

Journal:	*BMJ Open*
Manuscript ID	bmjopen-2020-040898.R2
Article Type:	Original research
Date Submitted by the Author:	29-Jul-2020

Complete List of Authors:	Price, Stephen; Cambridge University, Neurosurgery Division, Dept. Clinical Neurosciences<br>Joannides, Alexis; Cambridge University, Neurosurgery Division, Department of Clinical Neurosciences<br>Plaha, Puneet; John Radcliffe Hospital, Department of Neurosurgery<br>Afshari, Fardad; University Hospitals Coventry and Warwickshire NHS Trust, Department of Neurosurgery<br>Albanese, Erminia; University Hospitals of North Midlands NHS Trust, Department of Neurosurgery<br>Barua, Neil; North Bristol NHS Trust, Department of Neurosurgery<br>Chan, Huan Wee; University Hospital Southampton NHS Foundation Trust, Department of Neurosurgery<br>Critchley, Giles; Brighton and Sussex University Hospitals NHS Trust, Department of Neurosurgery<br>Flannery, Thomas; Royal Victoria Hospital<br>Fountain, Daniel; Salford Royal NHS Foundation Trust, Manchester Centre for Clinical Neurosciences<br>Mathew, Ryan; Leeds General Infirmary, Department of Neurosurgery<br>Piper, Rory; John Radcliffe Hospital, Department of Neurosurgery<br>Poon, Michael; The University of Edinburgh, Usher Institute<br>Rajaraman, Chittoor; Hull Royal Infirmary, Department of Neurosurgery<br>Rominiyi, Ola; Royal Hallamshire Hospital, Department of Neurosurgery<br>Smith, Stuart; Queen's Medical Centre, Department of Neurosurgery<br>Solomou, Georgios; University Hospitals Coventry and Warwickshire NHS Trust, Department of Neurosurgery; University of Birmingham College of Medical and Dental Sciences<br>Solth, Anna; Ninewells Hospital, Department of Neurosurgery<br>Surash, Surash; Royal Victoria Infirmary, Department of Neurosurgery<br>Wykes, Victoria; Queen Elizabeth Hospital, Department of Neurosurgery<br>Watts, Colin; University of Birmingham, Institute of Cancer and Genomic Studies<br>Bulbeck, Helen; brainstrust<br>Hutchinson, Peter; Cambridge University, Academic Neurosurgery Division, Department of Clinical Neurosciences<br>Jenkinson, Michael; The Walton Centre NHS Foundation Trust, Department of Neurosurgery

**Primary Subject Heading**:	Oncology

Secondary Subject Heading:	Neurology, Surgery
Keywords:	Neurological oncology < ONCOLOGY, Change management < HEALTH SERVICES ADMINISTRATION & MANAGEMENT, INFECTIOUS DISEASES

SCHOLARONE™
Manuscripts

I, the Submitting Author has the right to grant and does grant on behalf of all authors of the Work (as defined in the below author licence), an exclusive licence and/or a non-exclusive licence for contributions from authors who are: i) UK Crown employees; ii) where BMJ has agreed a CC-BY licence shall apply, and/or iii) in accordance with the terms applicable for US Federal Government officers or employees acting as part of their official duties; on a worldwide, perpetual, irrevocable, royalty-free basis to BMJ Publishing Group Ltd ("BMJ") its licensees and where the relevant Journal is co-owned by BMJ to the co-owners of the Journal, to publish the Work in this journal and any other BMJ products and to exploit all rights, as set out in our licence.

The Submitting Author accepts and understands that any supply made under these terms is made by BMJ to the Submitting Author unless you are acting as an employee on behalf of your employer or a postgraduate student of an affiliated institution which is paying any applicable article publishing charge ("APC") for Open Access articles. Where the Submitting Author wishes to make the Work available on an Open Access basis (and intends to pay the relevant APC), the terms of reuse of such Open Access shall be governed by a Creative Commons licence – details of these licences and which Creative Commons licence will apply to this Work are set out in our licence referred to above.

Other than as permitted in any relevant BMJ Author's Self Archiving Policies, I confirm this Work has not been accepted for publication elsewhere, is not being considered for publication elsewhere and does not duplicate material already published. I confirm all authors consent to publication of this Work and authorise the granting of this licence.

For peer review only

Impact of COVID-19 Pandemic on Surgical Neuro-oncology Multi-Disciplinary Team Decision Making – A National Survey (COVID-CNSMDT Study)

Stephen J Price[1], Alexis J Joannides[1], Puneet Plaha[2], Fardad T Afshari[3], Erminia Albanese[4], Neil U Barua[5], Huan Wee Chan[6], Giles Critchley[7], Thomas Flannery[8], Daniel M Fountain[9], Ryan Mathew[10], Rory J Piper[2], Michael TC Poon[11], Chittoor Rajaraman[12], Ola Rominiyi[13], Stuart Smith[14], Georgios Solomou[3,15], Anna Solth[16], Surash Surash[17], Victoria Wykes[18], Colin Watts[18], Helen Bulbeck[19], Peter Hutchinson[1] and Michael D Jenkinson[20]

On behalf of the COVID-CNSMDT study group

[1]Neurosurgery Division, Dept. Clinical Neurosciences, University of Cambridge, Cambridge CB2 0QQ; [2] Dept of Neurosurgery, John Radcliffe Hospital, Oxford OX3 9DU; [3] Dept of Neurosurgery, University Hospital Coventry, CV2 2DX; [4]Dept. of Neurosurgery, University Hospital of North Midlands, Stoke on Trent, ST46QG; [5]Dept. of Neurosurgery, North Bristol NHS Trust, Bristol BS10 5NB; [6]Dept of Neurosurgery, Wessex Neurological Centre, Southampton SO16 6YD; [7]Dept of Neurosurgery, Brighton and Sussex University Hospitals NHS Trust, Brighton BN2 5BE; [8]Dept of Neurosurgery, Royal Victoria Hospital, Belfast BT12 6BA; [9] Manchester Centre for Clinical Neurosciences, Salford Royal NHS Foundation Trust, Salford M6 8HD, [10]Dept of Neurosurgery, General Infirmary at Leeds, Leeds LS1 3EX; [11]Usher Institute, University of Edinburgh, EH16 4SB; [12]Dept of Neurosurgery, Hull Royal Infirmary, Hull HU3 2JZ; [13]Dept of Neurosurgery, Royal Hallamshire Hospital, Sheffield S10 2JF; [14]Dept of Neurosurgery, Queen's Medical Centre, Nottingham NG7 2UH; [15]University of Birmingham, Birmingham B15 2TT; [16]Dept of Neurosurgery, Ninewells Hospital and Medical

For peer review only - http://bmjopen.bmj.com/site/about/guidelines.xhtml

School, Dundee DD1 9SY; [17]Dept of Neurosurgery, Royal Victoria Infirmary, Newcastle upon Tyne NE1 4L; [18]Dept of Neurosurgery, Queen Elizabeth Neuroscience Centre, Birmingham B15 2TB; [19]Brainstrust, Cowes, Isle of Wight PO31 7QG; [20]Dept of Neurosurgery, University of Liverpool & The Walton Centre NHS Trust, Liverpool, L9 7LJ.

For peer review only

Corresponding Author:

Mr Stephen J Price

Hon. Consultant Neurosurgeon

Department of Clinical Neurosciences,

Division of Neurosurgery,

Box 167 Cambridge Biomedical Campus,

Cambridge CB2 0QQ.

E-mail: sjp58@cam.ac.uk

Tel: 01223 746455

WORD COUNT: 1848 words

For peer review only - http://bmjopen.bmj.com/site/about/guidelines.xhtml

ABSTRACT:

Objectives: Pressures on healthcare systems due to COVID-19 has impacted non-COVID patients with surgery disproportionally affected. This study aims to understand the impact on the initial management of patients with brain tumours by measuring changes to normal multidisciplinary team (MDT) decision making.

Design: A prospective survey performed in UK neurosurgical units performed from 23rd March 2020 until 24th April 2020.

Setting: Regional Neurosurgical Units outside London (as the pandemic was more advanced at time of study).

Participants: Representatives from all units were invited to collect data on new patients discussed at their MDT meetings during the study period. Each unit decided if management decision for each patient had changed due to COVID-19.

Primary and Secondary Outcome measures: Primary outcome: number of patients where the decision to undergo surgery changed compared to standard management usually offered by that MDT. Secondary outcome measures included – changes in surgical extent, numbers referred to MDT, number of patients denied surgery not receiving any treatment and reasons for any variation across the UK.

Results: 18 units (75%) provided information from 80 MDT meetings that discussed 1221 patients. 10.7% of patients had their management changed – the majority (68%) did not undergo surgery and more than half of this group not undergoing surgery had no active treatment. There was marked variation across the UK (0-28% change in management).

For peer review only

Units that did not change management could maintain capacity with dedicated oncology lists. Low volume units were less affected.

Conclusion: COVID-19 has had an impact on patients requiring surgery for malignant brain tumours, with patients receiving different treatments – most commonly not receiving surgery or any treatment at all. The variations show dedicated cancer operating lists may mitigate these pressures.

Study Registration: This study was registered with the Royal College of Surgeons of England's COVID Research Group (https://www.rcseng.ac.uk/coronavirus/rcs-covid-research-group/).

STRENGTHS AND LIMITATIONS OF THIS STUDY

- This is a National Survey that covers 75% of the UK units outside London providing a view of the impact COVID-19 had on the management of patients with brain tumours across the UK.
- Our data is based on 1221 patients.
- Data collection occurs two weeks before and two weeks after the peak of COVID-19 infections.
- We have not collected patient-level data so can't assess what happens to individual patients.
- We have only looked at surgical management and don't have data on oncology treatment.

For peer review only - http://bmjopen.bmj.com/site/about/guidelines.xhtml

BACKGROUND:

The COVID-19 pandemic has caused an unprecedented threat to healthcare delivery worldwide. Hospitals dealing with large numbers of patients requiring critical care have redeployed staff and converted operating rooms into intensive care units to cope with infected patients. This has had a marked impact on services and patient care for non-COVID-19 patients. Surgery has been disproportionately affected and requests to maintain urgent cancer surgery requires careful patient triage.[1-2]

In the UK adult brain tumours are managed across 30 regional neurosurgical centres, 24 of these are outside London. All new brain diagnoses are discussed in a multidisciplinary team meeting (MDT) that decides the optimal treatment for each patient. Each MDT will review a range of tumours from non-malignant or slow growing (low-grade) tumours whose initial treatment could be safely deferred, to the malignant tumours that are characterised by rapid growth and patient deterioration without treatment in a matter of weeks. Surgery is the main initial treatment for most patients – it can cure non-malignant tumours, remove the bulk of malignant tumours and is the only way of providing tumour tissue for pathological and molecular diagnosis to guide further treatment. Although surgery is not curative in malignant tumours it does prolong good quality survival. National guidelines recommended that surgery for malignant tumours should continue during the COVID-19 pandemic providing adjuvant oncology treatment is available.[3] The role of the MDT is critical to good patient management.

For peer review only - http://bmjopen.bmj.com/site/about/guidelines.xhtml

We undertook a national survey to evaluate the impact of COVID-19 on neurosurgical oncology services and to explore differences and variations in MDT decisions compared to the pre-COVID-19 era to investigate if patients were denied access to surgery.

For peer review only

For peer review only - http://bmjopen.bmj.com/site/about/guidelines.xhtml

METHODS:

Project leads from each of the UK neurosurgical units were identified to prospectively collect information from their weekly neuro-oncology MDT between 23rd March and 20th April 2020. London units were excluded as the pandemic peak was more advanced at the time of this study. Ethical approval was not required as no patient specific information was collected.

The primary outcome measure in this study is the change in MDT decision making. This was determined by each MDT. At each MDT meeting the project lead would review the decisions made for every patient with the rest of the team and decide whether this decision had differed from their normal practice due to COVID.

At each MDT meeting the following data were recorded:

- Total number of new patients discussed
- Number of patients where the MDT felt their initial/surgical treatment was different to their standard management due to COVID-19
  - Number of patients who did not receive surgery or whose surgical treatment intent was changed (e.g. biopsy rather than resection)
    - From the group that did not undergo surgery, the number of the patients who did not receive any active treatment (i.e. supportive care only).
  - Number of patients who had undergone surgery for a malignant tumour (defined as high grade glioma, metastatic tumour or other, rarer malignant tumour)

For peer review only - http://bmjopen.bmj.com/site/about/guidelines.xhtml

Variations in Decision Making

Data were compared to a baseline MDT workload from the 4 weeks of February (3rd – 28th February 2020) to provide information on the impact COVID-19. To explore the variations in MDT decision making, funnel plots for the proportion of cases discussed where management was altered were generated using Microsoft Excel, with 95% and 99.8% control limits calculated using the Wilson Score method for binomially distributed variates,[4]. Project leads were invited to comment with free text on how their unit responded to COVID-19. Using this free text we developed 3 groups that arranged units based on common expressed themes of how they managed to mitigate the challenges of COVID-19.

Statistical Analysis

Statistical analysis was performed using IBM SPSS 26. All variables were assessed for normality using a 1-sample Kolmogorov–Smirnov test. Changes in weekly mean number of new patients discussed at MDT meetings and operations for malignant tumours was assessed using paired t-test. Correlation of these variables with changes of MDT decision rates was made using a Pearson correlation.

Patient Involvement

Patients/the public were not involved the concept or design of this study as this came about from concerns among professionals at the services they were providing patients. A

For peer review only - http://bmjopen.bmj.com/site/about/guidelines.xhtml

patient/public representative was appointed to the study group during project set up to comment on the results.

For peer review only

For peer review only - http://bmjopen.bmj.com/site/about/guidelines.xhtml

RESULTS:

18 of the 24 eligible neurosurgical units (75%) participated in the study. 1221 new patients were included from 80 MDT meetings. In all units the functioning of the MDT changed with 8 units (33%) moving to fully video conferencing, all of the other units would limit attendance to a smaller number of senior representatives of different specialities (e.g. neurosurgery, oncology, radiology etc). 4 units (17%) reduced the duration of the meeting.

Comparing activity to baseline, there was a significant reduction in the number of new patients discussed from a mean weekly baseline (Table 1). 131 patients (10.7%) had a change in their initial management due to COVID-19. Of these, 15 (11.5%) had a change of surgical intent and 90 (68%) had no surgery at all. 47 patients (52% of those decided for no surgery) were decided for best supportive care. Information from project leads suggest the majority of these patients were elderly and had poor performance status. Other reasons for not operating were patients with low-grade gliomas or meningiomas whose surgery was deferred until after the critical phase of the pandemic.

The change in initial management varied between centres and ranged from 0 to 28%. This did not correlate with either the change in number of weekly operations (R=-0.03; P=0.91) nor number of patients discussed at an MDT meeting (R=-0.29; P=0.29). The funnel plot identified three groups of units (Figure 1). Group A was smaller units with low numbers of new brain tumour patients (mean = 7.4, 95%CI [4.1 – 10.6]) and low rates of management changes. Project leads commented that COVID had less impact on their units or were able to cope with the numbers of referrals. The group included a unit whose numbers decreased

For peer review only - http://bmjopen.bmj.com/site/about/guidelines.xhtml

from 28.5 to 13.6 per week (52% reduction). Group B was larger units (mean weekly new patients = 34.1, 95% CI [20.9, 47.2]) that allocated dedicated oncology lists. This was achieved by a variety of methods – implementing daily oncology lists, managing patients as emergencies or using the private hospital sector to provide space to treat brain tumour patients. Group C was the group with the largest change in management. A common theme for this group was the lack of dedicated oncology lists – patients with malignant tumours competing for surgical theatre operating time with other urgent surgical cases.

Patient/public interpretation of results.

The results were examined by the Study Group's patient/public representative (HB). They commented that "*when you're diagnosed with a brain tumour, your whole life is not only upended but put on hold. At this point, patients and their families need certainty. To be denied surgery - the first line treatment - doesn't bear thinking about. This impacts on the whole of the patient pathway, as without biopsies and resections, nobody really knows what you are treating, or how much tumour you are treating. It undermines the whole of the personalised medicine agenda and this cohort of patients will be living with the consequences, but probably not for as long as they would have been if they had had surgery. The short-sighted decision to bypass brain tumour surgery is undoubtedly shortening patients' lives.*"

For peer review only - http://bmjopen.bmj.com/site/about/guidelines.xhtml

DISCUSSION

This national study has shown the impact COVID-19 has had on MDT decision making for malignant (gliomas and metastases) brain tumour patients. 10.7% of patients had their initial management changed, of which the majority (68%) did not undergo surgery. Over half of these patients had palliative care only. Surgery is the first line treatment in malignant brain tumours and extent of resection is the only modifiable prognostic factor.[5] Patients with glioblastomas, the commonest malignant primary tumour, have a very limited survival without treatment,[6] and surgical resection improves efficacy of adjuvant therapies and quality of life.[7]

Like other cancers, COVID-19 has impacted on referrals.[8] We found a 27% reduction in a number of patients discussed and operations performed for malignant tumours (26% reduction) (cf. Table 1). Initial symptoms of brain tumours are frequently non-specific and patients often present when they decompensate.[9-10] The reduction in new tumour referrals during this study may result in patients presenting later and with more extensive disease.

An interesting finding was the variation between units. Group analysis identified trends in units that successfully reduced the impact of COVID-19 on MDT management. Strategies to ensure that dedicated cancer lists continued were helpful in larger units. It is clear that where cancer patients were scheduled on operating lists with other urgent cases more substantial MDT management changes were needed to cope. Our findings complement

For peer review only - http://bmjopen.bmj.com/site/about/guidelines.xhtml

other studies that have shown that the use of the private sector during this COVID pandemic results in less disruption to services.[11]

The study does have limitations, principally in the design. The purpose was to provide a prospective, rapid 'snapshot' of changes of MDT decision making during the peak of the pandemic using high level data. As such we did not include the detail from individual patient data that would perhaps allow better explanation of individual variation between units. Instead our project leads, all busy clinicians dealing with the impact of the peak of the pandemic, were able to obtain data on patient numbers. Although the study may lack some scientific rigor, it nevertheless provides a narrative of the real-world experiences seen by neurosurgical units during the height of the pandemic in the UK. Although this survey covered 70% of units, not all geographical areas have been included (e.g. South West and London). We have not been able to include the COVID-19 infection rate across the country into our analysis since each neurosurgery unit serves a large geographical area. The dates of our survey, however, correspond to the peak in cases for every geographical region.

Our primary outcome measure was, by its nature, pragmatic. This introduces some subjectivity between units as to what they defined as a change in management. As most of these changes involved no surgery or a change in surgical intent, we believe project leads reported significant changes in management that this study sought to explore. Our study only analysed changes in surgical treatment, but we know there are also changes in adjuvant therapy, such as radiation dose and chemotherapy regimes. It was difficult to quantify these changes in our study since patients are often treated in oncology centres that

For peer review only - http://bmjopen.bmj.com/site/about/guidelines.xhtml

are separate from the neurosurgery centres. The main focus of our study was to look at the MDT decision making where decisions about adjuvant therapy were made to influence surgery as national guidance recommend surgery for patients with malignant tumours only where adjuvant therapy was available.

In conclusion, COVID-19 has had an impact on patients requiring surgery for malignant brain tumours – with 10.7% having a change of management due to COVID-19, most commonly by not having surgery and many having no active treatment at all. The variation of changes in decision making show that dedicated cancer operating lists may help to mitigate the pressures of COVID-19.

For peer review only

Additional Information:

• **Acknowledgements**

Members of the COVID-CNSMDT Study group that delivered this study are (* project lead)

Belfast: Thomas Flannery* and Nicola Johnson

Birmingham: Victoria Wykes*, Georgios Solomou, Yasir Chowdhury and Colin Watts

Brighton: Giles Critchley* and Mandy Lynch

Bristol: Neil Barua*, George Malcolm, Ven Iyer, Constantinos Charalambides and Chris Herbert

Cambridge: Stephen Price*, Alexis Joannides, Richard Mair, Thomas Santarius , Fiona Harris and Olivia Poulter

Coventry: Fardad Afshari*, Georgios Solomou and Sandeep Solanki

Dundee: Anna Solth* and Kismet Hossain-Ibrahim

Edinburgh: Michael Poon* and Paul Brennan

Hull: Chittoor Rajaraman *, Shailendra Achawal and Gerry O'Reilly

Leeds: Ryan Mathew*, John Goodden and Simon Thomson

Liverpool: Michael D Jenkinson*, Andrew R Brodbelt, Emmanuel Chavredakis, David DA Lawson and Jibril Farah

Manchester: Tina Karabatsou* and Daniel Fountain.

Newcastle: Surash Surash*, Syed Shumon and Damian Holliman

Nottingham: Stuart Smith*, Muhammed Zafar, William Sage and Murugan Sitaraman

Oxford: Rory Piper* and Puneet Plaha

Sheffield: Ola Rominiyi* and Yahia Al-Tamimi

Southampton: Huan Wee Chan* and Paul Grundy

Stoke-on-Trent: Erminia Albanese* and Ebere Ogbonnaya

• Authors' contributions

SJP, MDJ, PP, CW and *PJH* were involved in the study concept and design.

SJP, TF, VW, GC, NB, FA, AS, MP, RC, RM, MDJ, DF, SSu, SSm, GS, RP, OR, HWC and *EA* were involved in collecting data from each site and guarantee the data at each site.

SJP and AJ were involved in data analysis.

HB provided the patient perspective on our findings.

SJP wrote the first draft of the paper and *all authors* were involved in revising the paper and have approved the final paper.

• Ethics approval and consent to participate

For peer review only - http://bmjopen.bmj.com/site/about/guidelines.xhtml

As this study did not collect personal data ethical approval was not required. It was registered as a service evaluation in each site.

• Consent for publication

No specific consent is required for publication.

• Data availability

Data access queries should be addressed to the corresponding author (SJP). All data is anonymised and individual unit level data is not available. Approval for data release will be made by SJP, MDJ, PP, CW and PJH who conceived the study.

• Conflict of interest

None of the authors have conflicts of interest.

• Funding

SJP is funded by a National Institute for Health Research (NIHR), (Career Development Fellowship). This paper presents independent research funded by the National Institute for Health Research (NIHR). The views expressed are those of the author(s) and not necessarily those of the NHS, the NIHR or the Department of Health and Social Care. MTCP is supported by Cancer Research UK Brain Tumour Centre of Excellence Award (C157/A27589).

For peer review only - http://bmjopen.bmj.com/site/about/guidelines.xhtml

REFERENCES

1. Al-Jabir A, Kerwan A, Nicola M, et al. Impact of the coronavirus (COVID-19) pandemic on surgical practice - Part 2 (surgical prioritisation). *Int J Sur* 2020 May 12, 10.1016/j.ijsu.2020.05.002.

2. Stevens S, Pritchard A. Important and urgent – next steps on NHS response to COVID-19. https://www.england.nhs.uk/coronavirus/wp-content/uploads/sites/52/2020/03/urgent-next-steps-on-nhs-response-to-covid-19-letter-simon-stevens.pdf (accessed March 2020).

3. The Society of British Neurological Surgeons. Adult Neuro-Oncology service provision during COVID-19 outbreak. https://www.sbns.org.uk/index.php/download_file /view/1646/1224/416/1224/. (accessed March 2020).

4. Spiegelhalter DJ. Funnel plots for comparing institutional performance. *Statistics in medicine* 2005;24(8):1185-1202.

5. Brown TJ, Brennan MC, Li M, et al. Association of the extent of resection with survival in glioblastoma: a systematic review and meta-analysis. *JAMA Oncol.* 2016;2(11):1460-1469.

6. Brodbelt A, Greenberg D, Winters T, et al. Glioblastoma in England: 2007-2011. *Eur J Cancer* 2015;51(4):533-542.

For peer review only - http://bmjopen.bmj.com/site/about/guidelines.xhtml

7. Stummer W, van den Bent MJ, Westphal M. Cytoreductive surgery of glioblastoma as the key to successful adjuvant therapies: new arguments in an old discussion. *Acta Neurochir (Wien)* 2011;153(6):1211-1218.

8. Dinmohamed AG, Visser O, Verhoeven RHA, et al. Fewer cancer diagnoses during the COVID-19 epidemic in the Netherlands. *The Lancet Oncology* 2020 Apr 30. doi 10.1016/s1470-2045(20)30265-5.

9. Walter FM, Penfold C, Joannides A, et al. Missed opportunities for diagnosing brain tumours in primary care: a qualitative study of patient experiences. *Br J Gen Pract* 2019;69(681):e224-e235.

10. Abel GA, Shelton J, Johnson S, Elliss-Brookes L, et al. Cancer-specific variation in emergency presentation by sex, age and deprivation across 27 common and rarer cancers. *Br J Cancer* 2015;112(Suppl 1):S129-136.

11. Jean WC, Ironside NT, Sack KD, et al. The impact of COVID-19 on neurosurgeons and the strategy for triaging non-emergent operations: a global neurosurgery study. *Acta Neurochir (Wien)* 2020;162(6):1229-40.

For peer review only

TABLE AND FIGURE LEGENDS

Table 1: Changes in workload between baseline and COVID-19 period. Malignant tumours refer to high grade gliomas, metastases and other rarer malignant tumours.

Figure 1: Funnel plot showing variation in number of patients whose management was changed due to COVID-19 vs. the number of patients discussed in MDTs over the study period. Standard deviation curves have been included. We have broken the data into 3 groups with different responses to the pandemic.

For peer review only

TABLE 1

	Baseline	During study period	Significance	Difference
**Number of new patients**	21.2 [95%CI 14.0 – 28.3]	15.3 [95%CI 10.0 – 20.6]	t=-4.26, P=0.0005	↓27%
**Number of operations for malignant tumours**	4.1 [95%CI 3.2 – 5.0]	3.0 [95%CI 2.0 – 4.1]	t=-2.76, P=0.01	↓26%

For peer review only

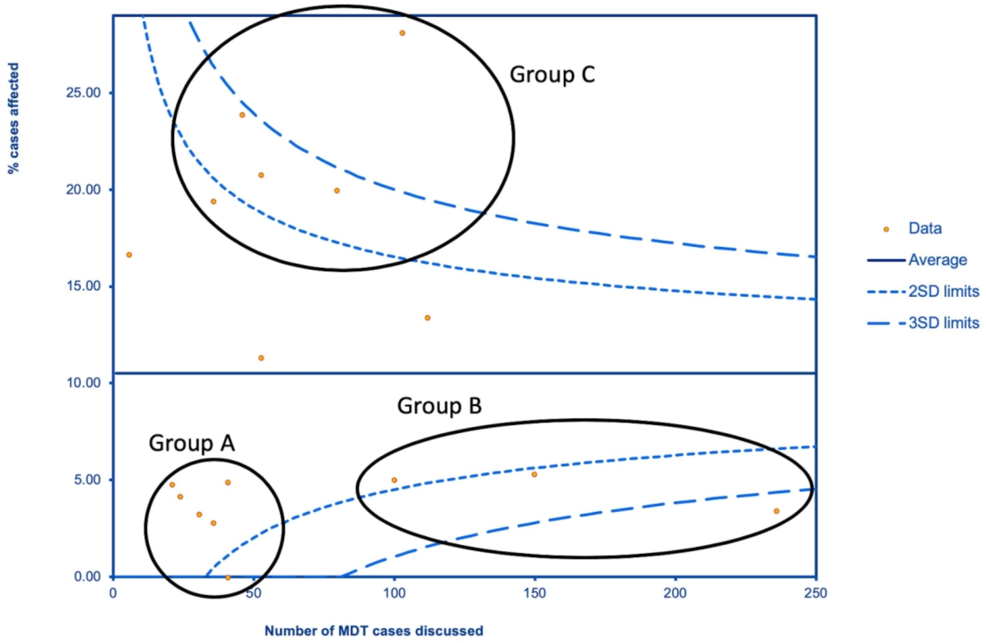

FIGURE 1: Funnel plot showing variation in number of patients whose management was changed due to COVID-19 vs. the number of patients discussed in MDTs over the study period. Standard deviation curves have been included. We have broken the data into 3 groups with different responses to the pandemic.

137x90mm (600 x 600 DPI)
